# Perceived Situational Appropriateness as a Predictor of Consumers' Food and Beverage Choices

**DOI:** 10.3389/fpsyg.2019.01743

**Published:** 2019-07-31

**Authors:** Davide Giacalone, Sara R. Jaeger

**Affiliations:** ^1^University of Southern Denmark, Odense, Denmark; ^2^The New Zealand Institute for Plant & Food Research Ltd., Auckland, New Zealand

**Keywords:** item-by-use, situational appropriateness, context, usage situation, food choice, product performance

## Abstract

This research investigated whether perceived situational appropriateness (defined as the degree of fit between product and intended usage situations) is predictive of consumer choices for foods and beverages, on the theoretical premise that intended usage situation acts as a frame of reference in orienting choices. Extant research on the topic, though suggestive of a link, is very limited in scope and almost completely lacking with regards to choice behavior (as opposed to other aspects, such as food acceptability or intake). To address the hypotheses, data collected in a series of 15 experiments (*N* = 2,813 consumers in total)—covering a wide range of product categories and usage situations—are presented. In all studies, participants evaluated a set of stimuli varying with respect to perceived appropriateness (low to high), and evaluated each stimulus either monadically using a choice likelihood scale or by performing a discrete choice task. Regression analyses from all studies consistently indicated that perceived appropriateness significantly predicted choice response. The results were robust with respect to variation in product category and experimental protocol and, overall, strongly support the notion that appropriateness can provide a simple yet powerful (in some case accounting for over 50% of variance) predictor of consumer choice. Effect sizes varied substantially: perceived appropriateness explained from a minimum of 3% to over 65% of variance in consumer choice, and this variation was linearly related to the degree of product heterogeneity in the product sets. This research also investigated possible moderators of the link between appropriateness and choice, by relating the results to consumers' product familiarity and involvement. While both traits significantly (and positively) affected choice, they did not interact with appropriateness. Possible explanations for these results, as well as other possible candidate moderators to explore in future research, are highlighted.

## 1. Introduction

The difficulty of explaining consumer behavior on the basis of product or individual consumers' characteristics has long been recognized in the consumer research literature, prompting a number of authors to devote attention to situational influences on consumer behavior (Sandell, 1968; Belk, 1975; Lai, 1991; Dubow, 1992; Warlop and Ratneshwar, 1993; Meiselman, 2008).

The theoretical premise for this line of work is that consumers' perceptions of products rarely occurs in isolation but, rather, relative to some frame of reference. The usage situation of a product is an ecological factor that can help define consumers' ends or goals, and thus orient their choices toward “situationally appropriate” solutions (Lai, 1991; Ratneshwar and Shocker, 1991; Giacalone and Jaeger, 2016). One important implications of this viewpoint is that consumers differentiate products on the basis of the anticipated usage situations, rather than purely on the basis of their individually stable preferences (Sandell, 1968; Belk, 1975; Ratneshwar and Shocker, 1991; Giacalone, 2018).

From a cognitive standpoint, situational effects on consumer choices can be explained on the basis of the compatibility principle (Tversky et al., 1988; Tversky and Simonson, 1993), according to which individuals tend to select options that are superior on the most salient dimension. Anticipated usage situation can orient consumers' attention to product attributes relevant to fulfilling goals associated with it. To illustrate the point, imagine a consumer who wishes to purchase a bottle of wine out of *n* alternatives, which we assume they explicitly considers. In theory, the consumer could evaluate all relevant attributes (e.g., price, origin, vintage, grape variety, alcohol by volume, etc.), assign a subjective value to each and choose the product with the highest overall value. This hypothetical decision making process is essentially the familiar theory of rational choice based on individual preferences. Let us now assume that the same person is choosing between the same set alternative keeping in mind, say, that they have just decided to lose weight. In such a situation, the compatibility principle suggests that they will be more likely to focus on the dimension made salient by the anticipated usage situation, which then provides a cognitively efficient “metric” for comparing alternatives. For instance, this consumer may equate alcohol content with caloric content, and choose the wine with the lowest alcohol content as the most appropriate given their current health goal. This second model of decision making process takes its point of departure into bounded rationality (the notion that consumers' information processing capabilities are limited and flawed) and assumes that consumer choices are often context-dependent (Tversky and Kahneman, 1986).

Following this line of research, a number of methodological approaches based on situational segmentation, i.e., on the identification of perceived product benefits across different situations (Dubow, 1992), have been developed. An analytical approach that is particularly relevant in the context of this research is the “substitution in use” (SIU) approach developed by Stefflre (1971) and later popularized by other authors (e.g., Ratneshwar and Shocker, 1991). In this approach, one typically asks consumers to evaluate the appropriateness of a set of product alternatives on a set of usage situations relevant to the product categories previously identified by the experimenter (e.g., through focus groups or based on prior research). The SIU approach focuses on the appropriateness of products with reference to a specific usage context, thus explicitly considering products as means to reach an end defined by a particular usage situation. In this paper, we define “usage context” as the anticipated usage situation for a product (Lai, 1991; Ratneshwar and Shocker, 1991), and “situational appropriateness” as the (subjectively evaluated) degree of fit between a product and a given usage context.

## 2. Background and Hypotheses

### 2.1. Situational Influences on Consumer Food-Related Behavior

Food and beverage consumption occurs in a wide variety of situations (Bisogni et al., 2007). Everyday experience tells us that specific usage situations direct our eating and drinking choices: for example, a consumer may highly appreciate a very complex wine when fine dining, but the same individual would be unlikely to choose it for a routine meal or a picnic. Not surprisingly, comparative studies have shown situational effects for food products are reported to be higher than for other product categories (Belk, 1975; Lai, 1991).

Perceived situational appropriateness is thus a highly relevant construct for explaining consumers' behavior with respect to food and beverages. While several studies have demonstrated that context can significantly affect important aspects of consumer experiences with foods and beverages, such as acceptance and consumption (Petit and Sieffermann, 2007), the influence of context on product choice has received markedly less attention. A notable exception is a study by Ariely and Levav (2000) in which the authors studied consumers food choice when dining with other people in a foodservice setting and found that, except for the individuals ordering first, choices in a group context may tend to reflect need for distinction rather than individual preferences. Another often cited paper is a study by Bell et al. who explored the effect of adding an Italian theme to a British restaurant and found that, while leaving the menu unaltered, consumers' choices were different from when the restaurant had its normal look, and were more oriented toward items perceived congruent with Italian cuisine (e.g., pasta items) (Bell et al., 1994).

Save for few exceptions, however, the paucity of information regarding the relationship between situational appropriateness and choice specifically has been remarked in the literature (Jaeger and Rose, 2008). Such information can potentially be obtained from revealed preference data (i.e., using observed choices in real markets) (as in Ariely and Levav, 2000), or by physically manipulating the consumption situation to fit specific research aims (as in Bell et al., 1994). However, both approaches are not very practical, especially if large consumer samples are to be included. Thus, stated choice experimentation—i.e., experiments in which participants make choices from sets of alternatives (e.g., Carson et al., 1994)—has been proposed as an alternative. Stated choice studies are regularly employed in the food consumer literature for the analysis of product variants on different attributes (e.g., Carlsson et al., 2007). Far less common is their application to study how consumers make choices conditional to the requirements of different situations, an approach which has been proposed for other product categories (Molin and Timmermans, 2010). Jaeger and Rose (2008) have noted the increasing need for situation–oriented choice experimentation in the food and beverage area, and Giacalone and Jaeger (2016) have proposed comparing situational appropriateness of use evaluations vis-à-vis stated choice data in order to understand the actual behavioral correlates of the appropriateness construct.

The present discussion has also considerable importance when taking a more applied perspective. In the food and beverage industry, hedonic responses (expressed as degree of liking or preference for a set of test products) have traditionally been the primary measure of a product's potential for market success (Giacalone, 2018, 2019), particularly in the context of new product development and line extensions. Although there is little doubt that preferences are an important determinant of food choice, previous research has shown that liking ratings in and of themselves are poor predictors of product success (Bell and Meiselman, 1995; Cardello et al., 2000). This has prompted an increasing search for alternative measures of product performance that can more effectively predict consumer food choices. The determination of the appropriateness of a product in different consumption situations is considered an important performance indicator to provide guidance in the product development process (Jaeger and Porcherot, 2017; Jaeger et al., 2017). To this end, several methodological approaches have been adopted, such as the Repertory Grid method (Jaeger et al., 2005), focus groups (Elzerman et al., 2013), personal interviews (Hartwell et al., 2016), and word associations (de Andrade et al., 2016).

Of particular relevance in the present paper is the “item-by-use” (IBU) method introduced by Howard Schutz as a complement to hedonic product tests (Schutz and Ortega, 1974; Schutz et al., 1975; Schutz, 1988). This approach consists in presenting a consumer with a list of possible consumption situations and asking him/her to indicate how well a product fit each of them.

While several authors have proposed that appropriateness evaluations using the IBU method may in fact be more predictive of consumer marketplace behavior than simple preferences (Marshall, 1993; Schutz, 1994; Bell and Meiselman, 1995), little attention has hitherto been devoted to empirically verify this proposition, and to quantify the relation between evaluations of situational appropriateness and relevant food-related behaviors, such as choice and consumption. In a rare exception, Sosa et al. (2005) found a strong relationships between degree of situational appropriateness and (self-reported) frequency of consumption of different seasoning sauces. Another similarly motivated study was published by Lai (1991), who investigated consumers' intention to adopt different versions of a canned beverage (Wulong tea) across different usage contexts, and found tentative indications that consumers were more willingly to adopt products perceived as more situationally appropriate. While these studies do suggest that consumers' choices may be predicted on the basis of perceived situational appropriateness, they are marred by being limited to a single product category and/or a single context, and based on a proxy (consumption frequency and intention adoption) rather than actual choice data. As a result, they do not completely shed light on the question of whether and to which extent consumer choices can be predicted from situational appropriateness judgments. Answering this question is the first aim of this paper.

### 2.2. Coenotropic vs. Individual Trade-Offs in Situational Appropriateness Evaluations

Situational appropriateness as a construct has a clear coenotropic nature, that is, it reflects rules learned through experience and cultural norms (Marshall, 1993; Rozin, 2006); the word “appropriateness” in and of itself suggests a normative aspect of how well a food fits the situation in which it is supposed to be consumed. Accordingly, appropriateness data reportedly have lower variance compared to more affective evaluations, such as preference or willingness-to-pay which place emphasis on subjective experiences (Giacalone, 2019). In this context, a question of theoretical and practical importance is which product–related and consumer–related factors can influence the effect of situational appropriateness on choice. For instance, how much of the variation in choice can be explained by appropriateness, and does this vary across different food and beverage categories?

A possible lens to understand these trade-offs is offered by the theory of situational strength (Mischel, 1977; Chang and Tseng, 2015), according to which individuals are more or less likely to conform to exogeneous (e.g., culturally determined) norms of behavior, rather than relying on their own preferences and personality traits, depending on different cues. The basic tenet of the theory is that when situational strength is high, people tend to refer to common norms and behave in a similar way, as the “normal” behavior is seen as strongly desirable. For example, a consumer may highly like wine but would rarely chose over coffee for breakfast. Conversely, when situational strength is low individuals are more likely to make idiosyncratic choices because there are few or no negative incentives to not follow the most normal behavior (and there may not even be clear indications as to what that would be). An example of low situational strength could be a consumer choosing a white wine over a red wine to go with a fine dining fish recipe, either because he is unaware of cultural conventions or because he is a wine connoisseur who feels confident in pairing a red wine with fish.

Consumers use different cues to infer how desirable a certain behavior is in a given situation (Chang and Tseng, 2015). When explicitly considering a set of product alternatives, we posit that the level of heterogeneity in the product set could in itself determine the level of situational strength: if alternative products are significantly different from one another, the salience of culturally appropriate roles would be enhanced (also as a way to reduce uncertainty), leading to a speedy identification of appropriate and inappropriate products (i.e., high situational strength). By contrast, if the product alternatives are similar enough, the cognitive processes by which consumers discern appropriateness would be more driven by their own preference and personality traits, thus exhibiting higher variability.

Generally, the magnitude of situational influences on consumer behavior differs considerably between studies (Belk, 1975; Quester and Smart, 1998), which can be attributed to differences inherent in the specific product categories considered, but also to different methodological approaches. This points to a need to go beyond single product studies in favor of large cross–product studies with common methodological approaches, thus providing a more solid basis for estimating the influence of situational appropriateness on consumer choices across varying experimental conditions.

Whilst we expect product heterogeneity to be important, consumers are also known to vary in their responsiveness to situational cues (Jaeger et al., 2019), and we therefore therefor also expect individual factors to explain part of the effect of appropriateness on choice. There is, at present, a need to better understand which individual characteristics influence evaluations of situational appropriateness (Quester and Smart, 1998; Giacalone et al., 2015; Giacalone and Jaeger, 2016; Jaeger et al., 2019). Even within the same cultural group, consumers may differ in their ability to infer perceived appropriateness, as they may have different expectations for, and experience with, different consumption situations (Bell and Meiselman, 1995; Quester and Smart, 1998). Nevertheless, very few studies have explicitly considered how individual characteristics mediate the effect of usage situations on consumer choices. In the already mentioned paper by Sosa et al. (2005) the authors noted a significant spread in individual responses; yet, segmenting consumers by relevant demographics did little to improve prediction, leading the authors to conclude that the effect of appropriateness on consumption is more likely to influenced by psychographic and behavioral aspects. Among the latter, product familiarity (amount of experience with a particular product category) and product involvement (how important a product is to a consumer) have been suggested as possible moderators of how usage situation affects consumers' food choices (Lai, 1991; Bell and Meiselman, 1995; Quester and Smart, 1998). Although both traits can be expected to exert an independent influence on food choice, as documented by numerous publications (e.g., Raudenbush and Frank, 1999; Hoek et al., 2011; Birch and Lawley, 2014; Behe et al., 2015), an argument can made that the more experience consumers have within a product category, the better they become at evaluating products (and their possible usages) independently of situational cues. Product familiarity and involvement, in other words, may lower situational strength. Conversely, consumers who have low familiarity and involvement with a product category, and who therefore cannot as easily rely on their experience to infer how well a product might perform, are more likely to use usage situation to orient their product choices. The role of product involvement as a moderator of situational influences has been previously explored in similarly motivated research. The reported results are mixed, but generally suggestive of a mitigating role of product involvement with respect to the importance of anticipated usage situations (Lai, 1991; Quester and Smart, 1998; Dodd et al., 2005). Product familiarity by contrast has received less attention. Recent findings by Schnurr et al. (2017) suggest that consumers who are familiar with products are less affected by the surrounding context when rating product quality, compared to consumers who lacked product familiarity. In this paper, we extend this line of consumer research literature by considering the role of product familiarity and involvement in moderating the effect of perceived appropriateness on choice.

### 2.3. Hypotheses

This section summarizes the literature review and presents specific hypotheses linked to the knowledge gaps highlighted above.

We started by reviewing previous research suggesting that food and beverage choice depends as much on the intended usage as it does on inherent product qualities. According to the compatibility principle, anticipated usage context acts as a frame of reference that can orient consumer choices by imposing constraints over the choice set and direct consumers toward situationally–appropriate solutions. The first hypothesis is, thus, formulated as follows:

**Hypothesis 1.** *When consumers make choices with a specific usage context in mind, a product with higher perceived situational appropriateness will be more likely to be chosen over one with lower appropriateness*.

Secondly, we argued that consumer evaluations of situational appropriateness will be influenced by several product characteristics, such as the product category, brand, packaging design, price points, and expectations regarding sensory properties. On the basis of the theory of situational strength, it is expected that consumer choices between competing alternatives will more likely defer to cultural norms regarding appropriateness in presence of large inter-product differences, whereas they will be more related to individual preferences for more homogeneous product sets. Hence, the second hypothesis is formulated as follows:

**Hypothesis 2.** *The degree to which appropriateness is predictive of product choice is positively related to the range of inter-product differences*.

Thirdly, we considered how the relationship between appropriateness and choice may be influenced by individual consumer characteristics, such as the amount of experience with a product category, and how these may make consumers more or less reliant on their preferences and attitudes in their choice behavior, relative to culturally determined rules for food and beverages. Based on extant literature, we expect situational appropriateness to be more salient for consumers who are less familiar and involved with a product category, because in such instances consumers cannot easily rely on experience or existing preferences, and are thus more likely to rely on external cues. Therefore, a third hypothesis is formulated as follows:

**Hypothesis 3.** *The influence of perceived appropriateness on choice is moderated by consumers' familiarity and involvement with the target product category, such that with increasing product familiarity and involvement, the effect of appropriateness on choice is decreased*.

## 3. Methods

### 3.1. Overview of Studies

All hypotheses were experimentally investigated in a series of 15 studies designed to collect appropriateness evaluations and choice data across a diverse range of food and beverage stimuli. A full overview of the studies conducted is given in Table 1, where the first column reports study IDs used to refer to individual studies throughout the paper.

**Table 1:** Overview of the 15 studies included in the paper (*N* = 2,813 in total).

**Study ID**	**Product category**	***N***	**Gender (% women)**	**Mean age (± St.Dev.)**	**Response format**	**Type of stimulus**	**No. of situations**
1	Breakfast items	112	66.1	42.9 ± 13.0	Choice likelihood	Names	1
2	Bakery items	102	51.0	42.6 ± 14.4	Choice likelihood	Names	1
3	Seafood	204	47.6	40.5 ± 14.8	Choice likelihood	Names	2
4	Beverages	102	51.0	40.6 ± 15.2	Choice likelihood	Names	3
5	Chocolate flavors	122	49.6	45.0 ± 13.3	Choice likelihood	Names	2
6a	Fruit	300	55.7	40.0 ± 10.9	Choice likelihood	Names	3
7a	White wine	300	60.0	39.5 ± 10.5	Choice likelihood	Images	3
8a	Beer	300	54.3	39.2 ± 10.8	Choice likelihood	Images	3
9a	Chocolate	300	59.0	39.0 ± 10.7	Choice likelihood	Images	3
10a	Kiwifruit	300	57.0	39.9 ± 10.9	Choice likelihood	Images	3
6b	Fruit	140	63.8	42.2 ± 13.4	Best-worst scaling	Names	3
7b	White wine	118	46.6	41.7 ± 12.7	Best-worst scaling	Images	3
8b	Beer	137	37.5	43.0 ± 13.1	Best-worst scaling	Images	3
9b	Chocolate	137	37.5	43.0 ± 13.1	Best-worst scaling	Images	3
10b	Kiwifruit	139	63.8	42.2 ± 13.4	Best-worst scaling	Images	3

The first set of studies (Studies 1–5) employed unbranded product names as stimuli, for which consumers were asked to evaluate perceived appropriateness for specific usage situations. Subsequently, they were asked to report their choice likelihood for each product in that specific usage situation, using a 7–pt likelihood scale. It was part of the research strategy that stimuli should cover a wide range of product categories varying in terms of their role in peoples' diet. Hence, focal product categories in Studies 1–5 included breakfast items, bakery items, seafood, beverages, and chocolate.

To further enhance the generalizability of the findings, a second set of five studies (Studies 6a–10a) was conducted, in which participants evaluated a set of stimuli chosen to represent fixed levels of situational appropriateness (Low, Medium, High) for target usage situations established using previously published data. Focal product categories in these studies included fruit, white wine, beer, chocolate bars, kiwifruit, breakfast items, and beverages. Three different usage contexts were evaluated for each study, and the stimulus format also varied across studies (product names vs. pictorial images of the product). Choice data were again obtained using a 7–pt likelihood scale. In this second set of studies, the influence of relevant individual traits (product involvement and familiarity) was also included in the experimental design. In addition to enhancing external validity by including a different stimulus format and additional product categories, Studies 6a–10a more closely resemble current practices in applied product testing, where appropriateness data collected from a consumer sample are used to generalize about a target population.

A final set of five studies (Studies 6b–10b) was conducted. These studies share with 6a–10a the same stimuli and usage situations but differed in that a discrete choice task (Best–Worst Scaling) was used to obtain choice data. The inclusion of this last set of studies was motivated by the need to assess the robustness of the results against methodological variations, while also checking the reproducibility of situation appropriateness data across different consumer samples.

### 3.2. Participants

All studies (*N* = 2,813 consumers in total) were conducted under central location tests (CLT) conditions in Auckland, NZ, with participants who were registered on a database maintained by a professional market research company. Inclusion criteria included being in the 18–65 age range and regular consumers of the focal products. A gender quota of at least 30% men was enforced in all studies. The specific age and gender composition for each study is reported in Table 1. All participants gave informed written consent and received cash compensation for their time. Ethical approval was sought and granted by the NZ Plant & Food research ethics committee prior to the recruitment.

### 3.3. Selection of Stimuli and Contexts

Stimuli employed in Studies 1–5 consisted of unbranded product names, a stimulus format previously employed effectively in this type of evaluations (Lai, 1991; Cardello et al., 2000; Giacalone, 2019). The complete list of stimuli and usage contexts used in Studies 1–5 is given in Table 2.

**Table 2:** List of stimuli used with corresponding level of appropriateness (IBU) in Studies 1–5.

**Study 1—Breakfast items**						
**Appropriateness**	**Context (*N* = 112)**					
	*For breakfast*	%				
**Highest**	Cereal	98.2^a^				
	Bacon and eggs	97.8^a^				
	Toast	96.4^a^				
	Waffles	70.5^b^				
	Muffins	61.6^c^				
	Scones	37.5^d^				
	Sandwich	23.2^e^				
	Sushi	12.5^f^				
	Macaroni and cheese	6.2^f,g^				
**Lowest**	French fries	2.7^g^				
**Q value**	606.9^***^					
**Study 2—Bakery items**						
**Appropriateness**	**Context (*N* = 102)**					
	*As part of a weekday evening meal*	%				
**Highest**	Garlick bread	97.0^a^				
	Wholemeal roll	81.4^b^				
	French stick (baguette)	74.5^bc^				
	Focaccia with olives	67.6^c^				
	Sourdough with caraway seeds	50^d^				
	White bread	46.1^d^				
	Ham and cheese muffin	17.6^e^				
	Chocolate croissant	12.7^ef^				
	Date scone	7.8^f^				
**Lowest**	Cinnamon and raisin bagel	5^f^				
**Q value**	409.1^***^					
**Study 3–Seafood**						
**Appropriateness**	**Context 1 (*N* = 99)**	**Context 2 (*N* = 105)**				
	*As part of a celebratory meal*	%	*In sushi or sashimi*	%		
**Highest**	Prawns	91.3^a^	Salmon	95.0^a^		
	Salmon	87.5^ab^	Tuna	92.1^a^		
	Scallops	83.6^ab^	Prawns	82.1^b^		
	Oysters	81.7^ab^	Snapper	63.4^c^		
	Snapper	77.9^bc^	Eel	47.5^d^		
	Mussels	70.2^cd^	Squid	47.5^d^		
	Squid	60.6^d^	Scallops	34.6^d^		
	Tuna	39.4^e^	Mussels	18.8^e^		
	Anchovies	23.1^f^	Anchovies	16.8^e^		
**Lowest**	Eel	17.3^f^	Oysters	13.9^e^		
**Q value**	334.3^***^	382.8^***^				
**Study 4—Beverages**						
**Appropriateness**	**Context 1 (*N* = 102)**		**Context 2 (*N* = 102)**		**Context 3 (*N* = 102)**	
	*For breakfast*	Mean	*For lunch*	Mean	*For dinner*	Mean
**Highest**	Hot beverage	6.6^a^	Water	6.9^a^	Wine	6.1^a^
	Water	6.5^ab^	Carbonated bev.	5.4^b^	Carbonated bev.	5.2^b^
	Juice	6.2^b^	Wine	4.5^c^	Hot bev.	4.3^c^
**Lowest**	Beer	1.4^c^	Milk	4.0^d^	Milk	3.3^d^
**F value**	727.8^***^		81.7^***^		56.1^***^	
**Study 5—Dark chocolate**						
**Appropriateness**	**Context 1 (*N* = 122)**		**Context 2 (*N* = 122)**			
	*After an enjoyable sit-down evening meal*	%	*At a picnic*	%		
**Highest**	Spicy Peppermint	58^a^	Burnt Coconut	53^a^		
	Burnt Coconut	45^ab^	Lemon & Cracked Pepper	30^b^		
	Lemon & Cracked Pepper	42^b^	Cardamom	25^b^		
	Plain Dark Chocolate	39^b^	Spicy Peppermint	25^bc^		
	Cardamom	38^b^	Plain Dark Chocolate	21^bcd^		
	Mediterranean Herbs	24^c^	Mediterranean Herbs	15^cd^		
	Fruit & Spice	21^c^	Fruit & Spice	14^d^		
**Lowest**	Wild Flowers	19^c^	Wild Flowers	12^d^		
**Q value**	85.4^***^		110.7^***^			

IBU data were elicited from consumers using a CATA response format. The table reports (1) the frequency (in percentage) with which each product was mentioned as appropriate for each context, (2) the test statistic for the Cochran's Q test (used to evaluate differences in appropriateness), (3) the p value for the test, and (4) the results of pairwise comparisons (within each context, frequencies which do not share the same superscript letter are significantly different at p < 0.05). In Study 4, appropriateness data were elicited with a 7-pt appropriateness scale. Differences between beverages were assessed using ANOVA. followed by Tukey's HSD test. n.s., not significant; ^*^p < 0.05; ^**^p < 0.01;

*** *p < 0.001*.

In addition to spanning diverse product categories, the selection of focal product categories in the Studies 1–5 was also informed by *H*_2_ in that we progressively narrowed the definition of the product category, from very broad (breakfast items in Study 1) to very specific (flavor concepts for a dark chocolate product in Study 5). Within each study, the process of identifying relevant stimuli items began by a brainstorming session among the authors who, in discussion with other consumer researchers agreed on an initial list of foods and beverages for each of the studies. The selection of usage contexts and target stimuli was done on the basis of pilot work to ensure that the products would span a large range of perceived situational appropriateness (from high to low) for each usage context. Table 2, where stimuli are ranked by perceived appropriateness, indicates that this was the case.

In the second set of Studies (Studies 6a–10a and 6b–10b), stimuli consisted either of product names or product images (Table 1), hence providing more comprehensive insights by including another stimulus format (pictorial images) that is very frequently employed in CTL tests (e.g., Jaeger et al., 2001; Giacalone and Jaeger, 2016). In these studies, stimuli were selected to represent specific levels of appropriateness based on pre-existing data (Giacalone et al., 2015; Giacalone and Jaeger, 2016). These datasets consist of situational appropriateness responses using the IBU method for a large number of use situations relevant to the product categories considered, collected on a larger set of stimuli. Usage situations were selected to span relevant dimensions of eating and drinking as identified in the literature (see e.g., Bisogni et al., 2007) and adopted a broad conceptualization of context including physical and temporal aspects of the situation, but also social surroundings, functional goals a consumer want to achieve and antecedent states (e.g., hunger, thirst, boredom, etc.) preceding consumption. Taking Study 6a as an example, the original dataset included 19 fruit types and 16 usage contexts: *As a healthy alternative to other snacks, As part of a dessert, As part of breakfast, Before bed, For children, For energy, For something a bit more sophisticated, For use in juices/smoothies, For variety in my fruit consumption, In a green salad, Something I can't resist, Something that is quick and easy, To eat on its own, To indulge myself*, *To share with others*, and *When riding in a car*. In the original study (Giacalone and Jaeger, 2016), participants were asked to evaluate each fruit name monadically and indicate which of the listed usage situations they considered it appropriate for using a check-all-that-apply (CATA) question format. Differences between product stimuli with regards to perceived appropriateness in different usage situations were assessed by Cochran's Q test, as commonly applied for this type of data (Giacalone and Jaeger, 2016). The three most discriminating contexts (in terms of Cochran's Q size) from each study were selected and, within each context, four stimuli representing, respectively, one highly appropriate product (High), two moderately appropriate products (Medium), and one situationally inappropriate product (Low). The complete list of stimuli and usage contexts used in Studies 6a–10a and 6a–10b is given in Table 3. As shown in Table 3, Studies 6a–10a and 6b–10b included the exact same stimuli and usage situations, but were conducted using different choice elicitation methods, as explained in section 3.4.

**Table 3:** List of stimuli used with corresponding level of appropriateness (Low, Medium, High) for each of the target contexts in Studies 6a–10a and 6b–10b.

**Studies 6a and 6b—Fruit Names (Giacalone and Jaeger**, **2016****)**						
**Appropriateness**	**Context 1 (*N* = 246)**		**Context 2 (*N* = 246)**		**Context 3 (*N* = 246)**	
	*As a healthy alternative to other snacks*	%	*As part of a dessert*	%	*For breakfast*	%
**High**	Apples	48^a^	Raspberries	49.1^a^	Bananas	48^a^
**Medium**	Gooseberries	32.5^b^	Gooseberries	32.9^b^	Passionfruit	31.3^b^
**Medium**	Tamarillo	28.8^b^	Oranges	31.3^b^	Oranges	30.8^b^
**Low**	Dragonfruit	15.8^c^	Dragonfruit	17.9^c^	Lychees	15.4^c^
**Q value**	61.0^***^		54.2^***^		58.5^***^	
**Studies 7a and 7b—White wine (Giacalone and Jaeger**, **2016****)**						
**Appropriateness**	**Context 1 (*N* = 112)**		**Context 2 (*N* = 112)**		**Context 3 (*N* = 112)**	
	*To drink with lunch*	%	*For a special occasion*	%	*With cakes and desserts*	%
**High**	Brancott Flight	59.8^a^	Lindauer	79.4^a^	Seifried Riesling	59.8^a^
**Medium**	Matawehero Gewurtztraminer	41.1^b^	Brancott Pinot Gris	41.1^b^	Lindauer	25.9^b^
**Medium**	Clearskin Pinot Gris	33.9^b^	Matawehero Gewurtztraminer	37.5^b^	Brancott Flight	21.4^b^
**Low**	Seifried Riesling	17^c^	Clearskin Pinot Gris	19.7^c^	Aronoui	9.8^c^
**Q value**	65.8^***^		99.7^***^		79.8^***^	
**Studies 8a and 8b—Beer (Giacalone et al.**, **2015****)**						
**Appropriateness**	**Context 1 (*N* = 97)**		**Context 2 (*N* = 97)**		**Context 3 (*N* = 145)**	
	*At a pub*	%	*At a casual dining restaurant*	%	*Watching a rugby game on TV at home*	%
**High**	Tui	84.5^a^	Steinlager Pure	64.9^a^	Steinlager Classic	91^a^
**Medium**	Mac's Hop Rocker	69.1^b^	Moa	44.3^b^	Mac's Hop Rocker	55.9^b^
**Medium**	Moa	51.5^b^	BrewMoon Ale	33^b^	Crafty Beggars	52.4^b^
**Low**	BrewMoon Ale	25.8^c^	Waikato	5.2^c^	Hopwired	42.1^c^
**Q value**	32.0^***^		49.1^***^		110.6^***^	
**Studies 9a and 9b—Chocolate (Giacalone and Jaeger**, **2016****)**						
**Appropriateness**	**Context 1 (*N* = 192)**		**Context 2 (*N* = 192)**		**Context 3 (*N* = 192)**	
	*For baking/cooking*	%	*When walking/hiking*	%	*For children*	%
**High**	Dairy Milk	55.7^a^	Energy	62^a^	Crunchie	73.4^a^
**Medium**	Caramello	12^b^	Coconut Rough	23.4^b^	Mint Bubbly	53.6^b^
**Medium**	Crunchie	9.9^b^	Dark Bubbly	17.2^c^	Fruit & Nut	35.9^c^
**Low**	Mint Bubbly	4.2^c^	Raspberry Mousse	4.2^d^	Old Jamaica Rum'n'Raisin	4.7^d^
**Q value**	219.6^***^		205.5^***^		226.8^***^	
**Studies 10a and 10b—Kiwifruit (Giacalone and Jaeger**, **2016****)**						
**Appropriateness**	**Context 1 (*N* = 302)**		**Context 2 (*N* = 302)**		**Context 3 (*N* = 302)**	
	*In a lunch box*	%	*As a digestive aid*	%	*When I feel like something refreshing*	%
**High**	Green fleshed–Pericarp type 3	54.3^a^	Green fleshed–Pericarp type 1	35.4^a^	Yellow fleshed–Pericarp type 3	47.4^a^
**Medium**	Green fleshed–Pericarp type 2	44^b^	Yellow fleshed–Pericarp type 3	28.5^b^	Red fleshed–Pericarp type 1	30.1^b^
**Medium**	Yellow fleshed–Pericarp type 1	40.4^b^	Yellow fleshed–Pericarp type 2	25.2^b^	Red fleshed–Pericarp type 2	29.5^b^
**Low**	Pink fleshed–Pericarp type 1	27.2^c^	Pink fleshed–Pericarp type 2	14.9^c^	Pink fleshed–Pericarp type 1	25.5^c^
**Q value**	93.3^***^		72.6^***^		72.7^***^	

All appropriateness data were elicited using a CATA response format. The table reports (1) the frequency (in percentage) with which each product was mentioned as appropriate for each context, (2) the test statistic for the Cochran's Q test (used to evaluate differences in appropriateness), (3) the p value for the test (n.s., not significant; ^*^p < 0.05; ^**^p < 0.01;

*** *p < 0.001), and (4) the results of pairwise comparisons (within each context, frequencies which do not share the same superscript letter are significantly different at p < 0.05). As noted in the text, the IBU data for these set of studies were collected as part of previously published research (Giacalone et al., 2015; Giacalone and Jaeger, 2016), to which the reader is referred to for additional information as well as for the images of the products*.

In relation to *H*_2_, this second set of studies is also characterized by a progressive removal of heterogeneity in the stimuli. Namely, Study 6 (a/b) included product names for different types of fruit, differing in size, seasonal availability, etc. Stimuli in Studies 7 and 8 were images of commercially available white wines and beer, respectively, which included different brands and price points. The last two studies also used images as stimuli but removed brand and price variation. Specifically, Study 9 (Chocolate) included products from a single brand at a similar price point, while Study 10 (Kiwifruit) included unbranded product concepts.

### 3.4. Experimental Procedures

#### 3.4.1. Stated Choice Studies (Studies 1–5 and 6a–10a)

Studies 1–5 required participants to perform two tasks. The first task consisted in evaluating the appropriateness of each stimuli for a target usage situation, using a CATA question format. For example, in Study 1 participants would be presented with a list of potential foods (see list in Table 2) and asked to tick all the ones they perceived as appropriate for breakfast. The order in which stimuli appeared in the list was randomized across participants to prevent order bias associated with this type of questions (Ares et al., 2014). The frequency with which each product was “ticked” as appropriate (reported in Table 2) was taken as a proxy for perceived appropriateness at aggregate level. The evaluation of appropriateness was slightly different in Study 4 compared to the other studies, as in this case participants rated each stimulus on 7–pt scale ranging from “Never appropriate” to “Always appropriate.” Accordingly, Table 2 shows mean ratings for this study.

The second task required participants to evaluate each product and rate the likelihood that they would choose that particular product in a target situation. Participants were presented with the product names sequentially listed in an individual ballot sheet and asked to rate the likelihood that they would choose that product in each target situation on a 7–pt scale ranging from 1 = “Very unlikely” to 7 = “Very likely.” For studies that involved more than one target contexts (i.e., all but Studies 1 and 2), the process was then repeated for any remaining context(s). The order in which participants evaluated products (and contexts) followed a balanced design to reduce the impact of carry–over and position effects.

Studies 6a-10a followed the same format but only required participants to carry out the second task (choice likelihood) since stimuli in these studies used pre-defined levels of appropriateness established using existing data (Table 3). In this second set of studies, participants were first asked to think about (each of) the target context(s) and imagine they were experiencing that situation. As suggested in Hein et al. (2012), participants were required to briefly describe the situation they were imagine in a few sentences to make the task more immersive. Subsequently, participants were presented with each of the products and asked to rate the likelihood that they would choose that product in the situation they just described using a 7–pt scale ranging from 1 = “Definitely not choose” to 7 = “Definitely choose.” Studies 7a–10a employed images as stimuli and therefore a separate ballot sheet for each product was provided, again, following a balanced order design for products and contexts.

At the end of each study, participants answered some background questions. In addition to standard demographic and socio-economic questions, participants reported their frequency of consumption and their degree of product involvement for the target product category. An involvement scale developed and validated by Lockshin et al. (1997) for measuring product involvement of wine consumers was employed in all studies. Three Likert items from their scale were selected, based on high factor loadings (Quester and Smart, 1996): “To me, [product category] matters,” “I have a strong interest in [product category],” and “[Product category] is important to me.” Participants rated each item on a 7-point Likert scale (1 = “Disagree extremely,” 7 = “Agree extremely”) to provide a composite measure of product involvement (range: 3–21, with larger values denoting greater involvement). Frequency of consumption was recorded using ordinal scales ranging from “Daily” to “Once per month” for fruit (Study 6a), and from “More than once a week” to “About once per 6–12 months” for the other product categories.

#### 3.4.2. Best-Worst Scaling Studies (Studies 6b–10b)

Studies 6b–10b were conducted similarly to Studies 6a–10a, with which they shared stimuli and contexts, but instead required participants to evaluate stimuli against each other in a discrete choice task. Participants were presented with a target situation and a set of three stimuli, and asked to indicate the one they were most likely to choose. For example, the task for Context 3 (*For breakfast*) of Study 6b (Fruit) read as follows:

*Think about an occasion when you would eat fruit as part of breakfast. Clearly imagine you are experiencing this occasion, please consider the following three types of fruit. If you had to make a choice, please indicate the one you would be MOST likely to choose, and also indicate the one you would be LEAST likely to choose*.

For each context, a balanced incomplete block design was used to generate four sets of three products, each of them occurring an equal amount of time. Continuing with the example given above, the first set would include *Passionfruit*–*Bananas*–*Lychees*, the second would include *Bananas*– *Lychees*–*Oranges*, the third *Lychees*–*Oranges*–*Passionfruit*, and the fourth *Orange*–*Passionfruit*–*Bananas*. Since each study included three contexts (Table 2), each participant effectively completed 12 B–W choice tasks. While in all previous studies choice likelihood ratings were taken as the main response variable, in Studies 6b–10b this was the Best–Worst Score (B–W) associated with each product. This score was obtained by counting the number of times a product was considered “most likely to choose” minus the number of times it was considered “least likely to choose,” resulting in individual-level scales for each product that are easily comparable across all participants (Marley and Louviere, 2005; Jaeger et al., 2008). Since all studies included four products, each occurring three times, the B–W score ranged from +3 to −3^1^.

## 4. Results

### 4.1. Effect of Situational Appropriateness on Choice (*H*_1_)

The data analytical strategy was informed by the three hypotheses. Mean choice likelihood ratings for each stimuli were computed and plotted by appropriateness level on a context–by–context basis to enable a visual exploration of the results. To evaluate the effect of the situational appropriateness level on consumers' choice likelihood (*H*_1_), a two–way analysis of variance (ANOVA) using the following model was conducted:

(1) $$\begin{array}{l} Y_{ijk}=\mu +A_{i}+P_{j}+AP_{ij}+C_{k}+\varepsilon_{ijk} \end{array}$$

where *Y*_*ijk*_ is the *ijk*^*th*^ observation for choice likelihood score, μ is the grand mean, *A*_*i*_, *P*_*j*_ and *AP*_*ij*_ represent, respectively, the fixed effects of appropriateness level, product and their interaction, *C*_*k*_ represents the random effect of consumers, and ε_*ijk*_ is the random error. Results from this analysis are reported in Table 4. As the table show, a significant effect of appropriateness level on consumers' choice was consistently found across all studies and all elicited contexts (*p* < 0.001 in all models). These results provide strong evidence that situational appropriateness is relevant for explaining consumers' product choices for food and beverages.

**Table 4:** ANOVA results showing effect of situational appropriateness level on product choice.

**Study**	**Product**	**Context**	**Df**.	***F***	***p***
1	Breakfast items	1. For breakfast	9	171.3	< 0.001
2	Bakery items	1. As part of a weekday evening meal	9	60.6	< 0.001
3	Seafood	1. As part of a celebratory meal	9	61.3	< 0.001
3	Seafood	1. In sushi or sashimi	8	82.3	< 0.001
4	Beverages	1. For breakfast	3	433	< 0.001
4	Beverages	2. For lunch	3	112.6	< 0.001
4	Beverages	3. For dinner	3	65.9	< 0.001
5	Chocolate flavors	1. (…) sit-down evening meal	7	48.9	< 0.001
5	Chocolate flavors	2. At a picnic	7	38.9	< 0.001
6a	Fruit	1. As a healthy alternative	2	556.9	< 0.001
6a	Fruit	2. As part of a dessert	2	257.2	< 0.001
6a	Fruit	3. For breakfast	2	1,518	< 0.001
7a	Wine	1. To drink with lunch	2	46.3	< 0.001
7a	Wine	2. For a special occasion	2	35.5	< 0.001
7a	Wine	3. With cakes and desserts	2	46.1	< 0.001
8a	Beer	1. At a pub	2	31.3	< 0.001
8a	Beer	2. At a casual dining restaurant	2	138.5	< 0.001
8a	Beer	3. Watching a rugby game on TV	2	32.4	< 0.001
9a	Chocolate	1. For baking/cooking	2	372.8	< 0.001
9a	Chocolate	2. When walking/hiking	2	219.6	< 0.001
9a	Chocolate	3. For children	2	299.1	< 0.001
10a	Kiwifruit	1. In a lunchbox	2	160.3	< 0.001
10a	Kiwifruit	2. As a digestive aid	2	158.8	< 0.001
10a	Kiwifruit	3. (…) something refreshing	2	170.1	< 0.001
6b	Fruit	1. As a healthy alternative	2	279.1	< 0.001
6b	Fruit	2. As part of a dessert	2	447.8	< 0.001
6b	Fruit	3. For breakfast	2	417.3	< 0.001
7b	Wine	1. To drink with lunch	2	133.5	< 0.001
7b	Wine	2. For a special occasion	2	118.4	< 0.001
7b	Wine	3. With cakes and desserts	2	70.9	< 0.001
8b	Beer	1. At a pub	2	47.9	< 0.001
8b	Beer	2. At a casual dining restaurant	2	193	< 0.001
8b	Beer	3. Watching a rugby game on TV	2	34.5	< 0.001
9b	Chocolate	1. For baking/cooking	2	727.6	< 0.001
9b	Chocolate	2. When walking/hiking	2	466.7	< 0.001
9b	Chocolate	3. For children	2	508.9	< 0.001
10b	Kiwifruit	1. In a lunchbox	2	245.1	< 0.001
10b	Kiwifruit	2. As a digestive aid	2	178.1	< 0.001
10b	Kiwifruit	3. (…) something refreshing	2	204	< 0.001

*The response variable is choice likelihood in Studies 1–5 and 6a–10a, and B–W score in studies 6b–10b. The number of degrees of freedom is the same in Studies 6a–10b because these studies used fixed appropriateness levels (Low, Medium, and High), whereas in the other studies appropriateness level is conflated with the individual products*.

The ANOVA model was supplemented by multiple linear regression to evaluate the degree to which appropriateness is predictive of choice likelihood, as well as the significance of the mean drop/increase in choice likelihood associated with varying levels of appropriateness. These results are presented in Table 5 for the first five studies and in Table 6 for the remaining ten studies.

**Table 5:** Regression results, Studies 1–5.

**Study**	**Context**	**b (Appropriateness)**	**Df**.	***F***	***p***	***R*^**2**^**
1 (Breakfast items)	1	+0.04^***^	1,1118	1,294	< 0.001	54%
2 (Bakery items)	1	+0.03^***^	1,1018	374.6	< 0.001	27%
3 (Seafood)	1	+0.04^***^	1,1048	278.1	< 0.001	21%
3 (Seafood)	2	+0.04^***^	1,988	367.9	< 0.001	27%
4 (Beverages)	1	+0.9^***^	1,562	1,125	< 0.001	67%
4 (Beverages)	2	+1.2^***^	1,562	321.4	< 0.001	36%
4 (Beverages)	3	+1.1^***^	1,562	188.2	< 0.001	25%
5 (Chocolate flavors)	1	+0.02^***^	1,966	22.4	< 0.001	2%
5 (Chocolate flavors)	2	+0.03^***^	1,966	37.4	< 0.001	4%

n.s., not significant; ^*^p < 0.05; ^**^p < 0.01;

*** *p < 0.001*.

**Table 6:** Regression results, Studies 6a–10a and 6b–10b.

**Study**	**Context**	**b (High)**	**b (Medium)**	**b (Low)**	**Df**.	***F***	***p***	***R*^**2**^**
6a (Fruit)	1	+6.0 ^***^	−2.9 ^***^	−3.4 ^***^	2,1197	365.6	< 0.001	38%
6a (Fruit)	2	+5.5 ^***^	−1.7 ^***^	−2.8 ^***^	2,1197	183.2	< 0.001	23%
6a (Fruit)	3	+5.9 ^***^	−1.8 ^***^	−3.2 ^***^	2,1197	209.3	< 0.001	26%
7a (Wine)	1	+4.4 ^***^	−0.5 ^***^	−1.1 ^***^	2,1197	32.6	< 0.001	5%
7a (Wine)	2	+4.6 ^***^	−0.7 ^***^	−0.9 ^***^	2,1197	25.3	< 0.001	4%
7a (Wine)	3	+4.3 ^***^	−0.6 ^***^	−1.2 ^***^	2,1197	34.0	< 0.001	5%
8a (Beer)	1	+3.7 ^***^	−0.3 ^*^	−0.9 ^***^	2,1197	17.8	< 0.001	3%
8a (Beer)	2	+4.4 ^***^	−1.2 ^***^	−2.0 ^***^	2,1197	93.0	< 0.001	13%
8a (Beer)	3	+4.0 ^***^	−0.6 ^***^	−0.8 ^***^	2,1197	17.5	< 0.001	3%
9a (Chocolate)	1	+4.8 ^***^	−2.1 ^***^	−2.8 ^***^	2,1197	204.6	< 0.001	25%
9a (Chocolate)	2	+5.1 ^***^	−1.6 ^***^	−2.4 ^***^	2,1197	130.3	< 0.001	18%
9a (Chocolate)	3	+4.7 ^***^	−1.2 ^***^	−3.0 ^***^	2,1197	217.2	< 0.001	26%
10a (Kiwifruit)	1	+5.2 ^***^	−0.7 ^***^	−2.0 ^***^	2,1197	83.5	< 0.001	12%
10a (Kiwifruit)	2	+5.2 ^***^	−0.6 ^***^	−1.8 ^***^	2,1197	68.2	< 0.001	10%
10a (Kiwifruit)	3	+4.8 ^***^	−1.3 ^***^	−1.5 ^***^	2,1197	59.3	< 0.001	9%
6b (Fruit)	1	+0.54^***^	−0.64^***^	−0.87^***^	2,2237	297.6	< 0.001	21%
6b (Fruit)	2	+0.64^***^	−0.75^***^	−1.00^***^	2,2237	477.5	< 0.001	30%
6b (Fruit)	3	+0.56^***^	−0.59^***^	−1.10^***^	2,2237	445.0	< 0.001	28%
7b (Wine)	1	+0.38^***^	−041^***^	−0.72^***^	2,1885	142.4	< 0.001	13%
7b (Wine)	2	+0.27^***^	−0.21^***^	−0.67^***^	2,1885	126.3	< 0.001	12%
7b (Wine)	3	+0.31^***^	−0.34^***^	−0.51^***^	2,1885	75.6	< 0.001	7%
8b (Beer)	1	−0.06^***^	+0.19^***^	−0.16^***^	2,2189	51.04	< 0.001	4%
8b (Beer)	2	+0.23^***^	−0.11^***^	−0.71^***^	2,2189	205.8	< 0.001	16%
8b (Beer)	3	+0.07^*^	0.00^*n*.*s*^	−0.29^***^	2,2189	36.8	< 0.001	3%
9b (Chocolate)	1	+0.68^***^	−0.72^***^	−1.28^***^	2,2189	775.8	< 0.001	41%
9b (Chocolate)	2	+0.56^***^	−0.57^***^	−1.11^***^	2,2189	497.7	< 0.001	31%
9b (Chocolate)	3	+0.48^***^	−0.40^***^	−1.12^***^	2,2189	542.6	< 0.001	33%
10b (Kiwifruit)	1	+0.36^***^	−0.29^***^	−0.85^***^	2,2221	261.4	< 0.001	18%
10b (Kiwifruit)	2	+0.17^***^	−0.03^*n*.*s*^	−0.64^***^	2,2221	189.9	< 0.001	14%
10b (Kiwifruit)	3	+0.44^***^	−0.49^***^	−0.80^***^	2,2221	217.5	< 0.001	16%

*n.s., not significant*;

* *p < 0.05*;

** *p < 0.01*;

*** *p < 0.001*.

**Figure 1:**
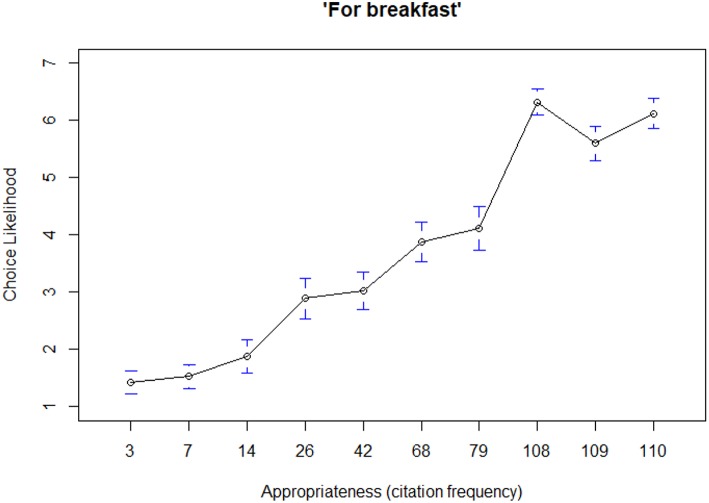
Mean choice likelihood ratings (7-pt scale) by level of product appropriateness for Study 1. Confidence intervals (95% are also shown).

Starting from Studies 1–5, the results showed a linear increase in choice likelihood with increasing level of appropriateness in all studies (Table 5), in line with our first hypothesis (*H*_1_). This conclusion was also supported by visual interpretation of the results. For example, Figure 1 shows mean choice likelihood ratings for all products used in Study 1. Inspecting the figure (note that the appropriateness citation data reported in the abscissa correspond to those reported in Table 5), one can see that there were three highly appropriate items (Cereals, Bacon and eggs, and Toast) that received the highest ratings for choice likelihood (averaging between 5.6 and 6.2 on a 7–pt scale), four moderately appropriate items (Waffles, Muffins, Scones, and Sandwich) that got a lower mean ratings (2.9−4.1), and three inappropriate items (Sushi, Mac and cheese, and French fries) that received the lowest ratings (1.4−1.9). Accordingly, the linear regression model for this dataset revealed that situational appropriateness significantly predicted choice likelihood and accounted for 54% of its variance (Table 5). Corresponding plots from the other studies (not shown here but available as Supplementary Material to this paper) all showed the same pattern.

On average, appropriateness ratings explained 29.2% of the variance in choice likelihood, although effect sizes (*R*^2^) varied substantially across studies, ranging from a minimum of 2% in Context 2 of Study 5, to a maximum of 67% in Context 1 of Study 4 (Table 5). Study 4 also showed a significant variation in effect sizes between contexts, as appropriateness explained 67% of the variance in choice in Context 1 (“For breakfast”), but only 36 and 25% for the other two contexts (“For lunch” and “For dinner,” respectively).

**Figure 2:**
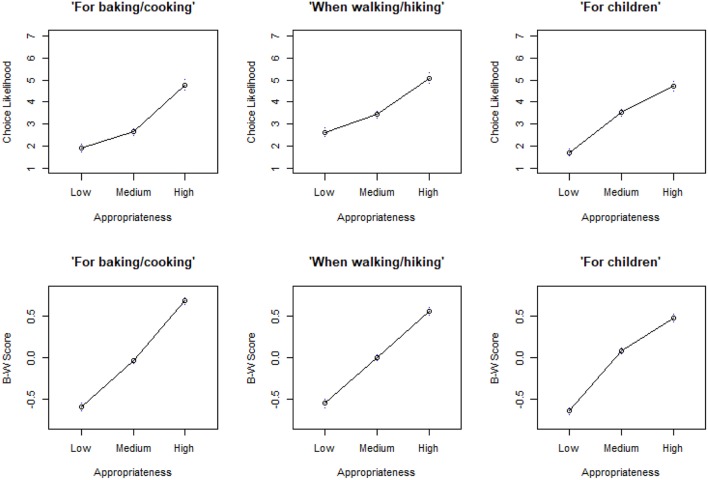
Mean choice likelihood ratings and B–W scores by level of appropriateness for Studies 9a (top) and 9b (bottom).

Regression results from the remaining ten studies are reported in Table 6. Recall that this second set of studies is different from the previous one, where appropriateness was estimated directly from the same population of consumer who performed the choice task, in that stimuli were chosen to represent fixed levels of appropriateness based on existing data obtained with a different (though comparable) consumer population. In order to better enable a comparison between ratings and discrete choice, the results are also visually plotted: an example can be seen in Figure 2 which shows results from Studies 9a and 9b (corresponding plots from all other studies are available as Supplementary Material to this paper). Generally, regression analyses showed that the high appropriateness significantly predicted choice across all studies and all contexts (Table 6), thus supporting the results obtained in the first five studies. As expected, a significant drop in mean choice likelihood/B–W score was observed when moving from the High to Medium appropriateness level, and again from Medium to Low (notice that the regression coefficients in Table 6 for the Low and Medium groups correspond to the difference in mean choice likelihood/B–W score compared to the High group). There were three instances where this did not hold completely true. In Study 8b, Context 1, the Medium appropriateness level is associated to a higher B–W score than the High level (see Supplementary Material). The reason for this is unclear but in-depth analyses showed that it was due to one of the two products used to represent the Medium appropriateness level (“Mac's Hop Rocker”) performing better than the product chosen as representing the High appropriateness level (“Steinlager Classic”). Since the local beer market had been undergoing significant changes in terms of product availability in recent years, and considering the time elapsed between collection of appropriateness data and the B–W experiments (about 1 year), we speculate that this result may be due to the rise in popularity of craft and micro-breweries (Giacalone et al., 2015; Cardello et al., 2016) and a change in consumers' perception of this particular product. In two other instances (Context 3 of Study 8b, and Context 2 of Study 10b) the Medium and High appropriateness were not differentiated, though both were significantly larger than the one for the Low level.

Aside from these minor exceptions, the regression results were fully in line with our hypothesis that appropriateness would be predictive of choice (*H*_1_); this was also fully supported by visual inspection of the results, where mean choice likelihood ratings and B–W scores can be seen as increasing with increasing appropriateness levels (as exemplified in Figure 2). Like in the first set of studies, a substantial variation in effect sizes was observed (3% < *R*^2^ < 41%, *Mean* = 17%). Within studies, differences on a context by context basis were also present. For example, in Study 6a the amount of explained variance in choice data explained by the regression model was 38% in Context 1, but dropped to 23% and 26% in Contexts 2 and 3. In both beer studies (8a and 8b), the *R*^2^ was substantially larger in Context 2 than in the other two contexts (Table 6). However, by and large variation in effect size appeared to be more related to the stimuli used than to the individual usage situations. Accordingly, a diagnostic ANOVA model using data from Table 6 confirmed a significant effect of “Study” on *R*^2^ [All studies: *F*_(9, 20)_ = 14.6, *p* < 0.001; Studies 6a–10a only: *F*_(4, 10)_ = 14.7, *p* < 0.001; Studies 6b–10b only: *F*_(4, 10)_ = 16.6, *p* < 0.001].

Inspection of Figure 2 and corresponding plots from all other studies indicated a remarkable consistency between results elicited using choice likelihood (6a–10a) and those obtained with a discrete choice task (6b–10b). Recall that a direct and straightforward comparison is made possible by the fact that stimuli and contexts in Study 6a–10a are identical to those in Studies 6b–10b. It was also noteworthy that, considering the five product categories that were in common across the two set of studies, both methods consistently produced the same ranking in terms of effect sizes—Chocolate (Studies 9a–9b), then Fruit (6a–6b), then Kiwifruit (10a–10b), then Wine (7a–7b), and finally Beer (8a–8b)—strongly indicating that the results were robust with respect to methodological variations in choice elicitation protocol.

### 4.2. Relationship Between Effect Sizes and Stimulus Heterogeneity (*H*_2_)

The results presented thus far indicated that appropriateness significantly affects choice. The direction of the effect was fully in line with our first hypothesis, but its size varied substantially across studies. This was in line with our second hypothesis, according to which appropriateness is predictive of choice should be positively related to product heterogeneity, i.e., to the degree of differences amongst the set of stimuli evaluated by the consumers (*H*_2_). To address this hypothesis, we considered effect sizes (expressed as adjusted *R*^2^) for each regression model relating appropriateness on choice likelihood for each individual usage situation across all studies, and the Cochran's Q test statistic for the corresponding usage context. The reason for using this as a metric for the degree of differences in appropriateness is that Cochran's Q tests the null hypothesis that the proportion of consumers ticking a product as appropriate would be equal for all products; conversely, the larger the Q value, the greater the deviation from the null hypothesis in at least one of the product. Effect sizes (*R*^2^) and Q values for individual usage contexts were plotted and the strength of their linear correspondence was used to address *H*_2_. Note that Study 4 is excluded from this analysis because the method for eliciting appropriateness ratings was slightly different as previously explained (Cf. 3.4).

**Figure 3:**
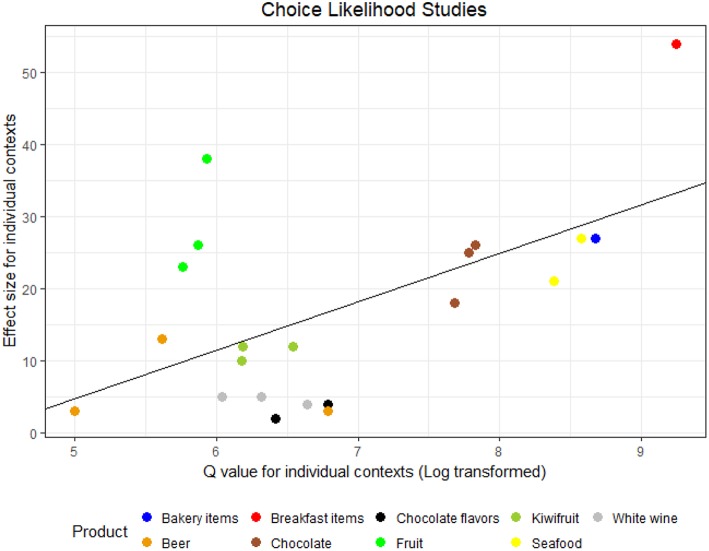
Size of the difference in appropriateness (Cochran's *Q*) plotted against effect size (*R*^2^) for studies 1–5 and 6a–10a, with superimposed line of best fit (*y* = −28.7 + 6.7*x, R*^2^ = 33%, *p* = 0.006).

**Figure 4:**
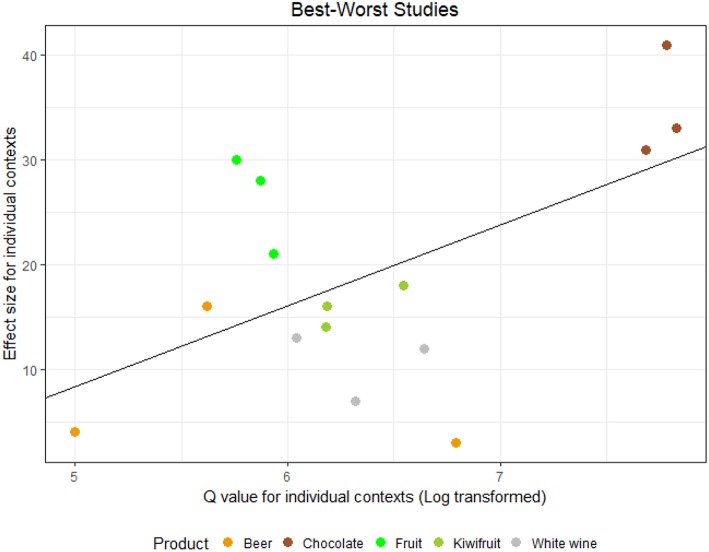
Size of the difference in appropriateness (Cochran's *Q*) plotted against effect size (*R*^2^) for studies 6b–10b, with superimposed line of best fit (*y* = −30.1 + 6.7*x, R*^2^ = 31%, *p* = 0.029).

These results are illustrated in Figures 3, 4 for, respectively, studies employing choice likelihood, and studies employing B–W scaling. Both figures show that as the degree of heterogeneity in the product set increases, so does the effect size in a roughly linear manner. Accordingly, correlational analyses indicated a moderate-to-strong [CL studies: *r*_(19)_ = 0.69, *p* =< 0.001; B–W studies: *r*_(13)_ = 0.63, *p* = 0.011] relationship between these two quantities.

Individual contexts in Figures 3, 4 are color–coded by product category (study), allowing further interpretation of the results. In general, albeit variation within studies exist, this is clearly less important than between studies variation as data points belonging to the same study tend to cluster together. This confirms the earlier intuition that the strength of the relationship between appropriateness and choice is more dependent on the product set, whereas it is relatively stable across contexts. Inspecting Figure 3 from right to left we can see the furthest point corresponds to Study 1 (Breakfast products), a study which employed widely different stimuli and spanned a very large range in appropriateness (*Q* = 606.9, cf. Table 2). Study 2 (Bakery items) and Study 3 (Seafood, both contexts) followed closely. Both studies were similar to Study 1 in terms of product differences and stimulus type (names). We then find Study 9a, which also featured very large differences in perceived appropriateness (*Q* > 200 in all contexts), despite employing a theoretically more homogeneous set of stimuli (images of chocolate bars from a single brand at a similar price point). Further we find a cluster of diverse studies employing either unbranded product concepts (Kiwifruit, Chocolate flavors) and product images (white wine), but all in the moderate range concerning product differences (65 < *Q* < 110).

The three contexts from Study 6a (fruit names) follow suit and appear to deviate somewhat from the other data points in Figure 3 as they feature some of the largest effect sizes (23% < *R*^2^ < 38%) but have limited difference in term of perceived appropriateness (54 < *Q* < 61). That this study is somewhat of an outlier is also suggested by the fact that removing it increased the correlation coefficient significantly (*r*_(16)_ = 0.91, *p* < 0.001). The data points from Study 8a (Beer images) close the sequence, in particular Context 1 which had the lowest product heterogeneity in terms of appropriateness (*Q* = 32) and correspondingly the smallest effect size.

Studies employing a B–W elicitation protocol showed an identical pattern and ranking in terms of product categories (Figure 4). Again, partial deviations from linearity were only observed in the study that employed fruit names as stimuli (Study 6b) and a corresponding increase in correlation coefficient could be obtained by removing those data points [*r*_(10)_ = 0.85, *p* < 0.001]. Taken collectively, these results appear in line with hypothesis *H*_2_ and indicate that the magnitude of the effect of appropriateness on choice increases with progressive increase of product heterogeneity following a linear, albeit imperfect, trajectory.

### 4.3. Effect of Consumer Involvement and Familiarity (*H*_3_)

Our third hypothesis considered the role of consumer traits expected to moderate the effect of perceived appropriateness on choice. To address *H*_3_, the role of product involvement and consumption frequency were assessed by analysis of co-variance (ANCOVA), i.e., by including these individual characteristics (numeric) as covariate together with appropriateness level (categorical) to predict choice. According to *H*_3_, a significant interaction between appropriateness and these two individual variables was expected. The results are presented in Table 7 which report both independent (main) and moderating (two-way interactions with appropriateness level) effects for the two covariates^2^.

**Table 7:** ANCOVA table showing main effect of covariates (involvement and frequency) on product choice as well as interactions with appropriateness in Studies 6a–10a.

**Study**	**Context**	**Effect**	**Df**.	***F***	***p***	***R*^**2**^**	**Δ*R*^**2**^**
**COVARIATE: INVOLVEMENT**							
6a (Fruit)	1	Involvement	1	55.5	< 0.001	41%	+3%
		Appropriateness: Involvement	2	0.1	0.920		
6a (Fruit)	2	Involvement	1	62.2	< 0.001	27%	+4%
		Appropriateness: Involvement	2	1.4	0.232		
6a (Fruit)	3	Involvement	1	118.8	< 0.001	32%	+6%
		Appropriateness: Involvement	2	1.1	0.335		
7a (Wine)	1	Involvement	1	3.4	0.066	6%	+1%
		Appropriateness: Involvement	2	5.0	0.006		
7a (Wine)	2	Involvement	1	0.6	0.439	4%	0%
		Appropriateness: Involvement	2	0.7	0.475		
7a (Wine)	3	Involvement	1	18.8	< 0.001	7%	+2%
		Appropriateness: Involvement	2	3.3	0.039		
8a (Beer)	1	Involvement	1	47.0	< 0.001	6%	+3%
		Appropriateness: Involvement	2	1.5	0.219		
8a (Beer)	2	Involvement	1	50.1	< 0.001	17%	+4%
		Appropriateness: Involvement	2	0.6	0.552		
8a (Beer)	3	Involvement	2	81.0	< 0.001	9%	+6%
		Appropriateness: Involvement	2	2.0	0.134		
9a (Chocolate)	1	Involvement	1	50.7	< 0.001	28%	+3%
		Appropriateness: Involvement	2	1.9	0.149		
9a (Chocolate)	2	Involvement	1	114.7	< 0.001	25%	+7%
		Appropriateness: Involvement	2	0.2	0.792		
9a (Chocolate)	3	Involvement	1	56.1	< 0.001	30%	+4%
		Appropriateness: Involvement	2	4.0	0.017		
10a (Kiwifruit)	1	Involvement	1	93.3	< 0.001	18%	+6%
		Appropriateness: Involvement	2	0.1	0.935		
10a (Kiwifruit)	2	Involvement	1	152.1	< 0.001	20%	+10%
		Appropriateness: Involvement	2	1.5	0.226		
10a (Kiwifruit)	3	Involvement	1	87.4	< 0.001	15%	+6%
		Appropriateness: Involvement	2	0.5	0.606		
**COVARIATE: FREQUENCY**							
6a (Fruit)	1	Frequency	1	31.1	< 0.001	39%	+1%
		Appropriateness: Frequency	2	0.6	0.552		
6a (Fruit)	2	Frequency	1	22.6	< 0.001	25%	+2%
		Appropriateness: Frequency	2	0.6	0.521		
6a (Fruit)	3	Frequency	1	35.6	< 0.001	28%	+2%
		Appropriateness: Frequency	2	0.5	0.576		
7a (Wine)	1	Frequency	1	1.0	0.303	5%	0%
		Appropriateness: Frequency	2	0.7	0.505		
7a (Wine)	2	Frequency	1	1.8	0.182	4%	0%
		Appropriateness: Frequency	2	1.7	0.189		
7a (Wine)	3	Frequency	1	0.3	0.589	5%	0%
		Appropriateness: Frequency	2	1.5	0.228		
8a (Beer)	1	Frequency	1	7.2	0.007	3%	0%
		Appropriateness: Frequency	2	0.5	0.597		
8a (Beer)	2	Frequency	2	15.1	< 0.001	15%	+2%
		Appropriateness: Frequency	2	1.2	0.287		
8a (Beer)	3	Frequency	1	8.7	0.003	3%	0%
		Appropriateness: Frequency	2	2.8	0.062		
9a (Chocolate)	1	Frequency	1	14.5	< 0.001	26%	+1%
		Appropriateness: Frequency	2	0.1	0.865		
9a (Chocolate)	2	Frequency	1	24.7	< 0.001	19%	+1%
		Appropriateness: Frequency	2	0.5	0.630		
9a (Chocolate)	3	Frequency	1	20.9	< 0.001	28%	+2%
		Appropriateness: Frequency	2	0.9	0.420		
10a (Kiwifruit)	1	Frequency	1	23.9	< 0.001	14%	+2%
		Appropriateness: Frequency	2	0.1	0.926		
10a (Kiwifruit)	2	Frequency	1	54.6	< 0.001	14%	+4%
		Appropriateness: Frequency	2	2.0	0.138		
10a (Kiwifruit)	3	Frequency	1	14.7	< 0.001	10%	+1%
		Appropriateness: Frequency	2	0.3	0.740		

*The last column (ΔR^2^) reports the change in explained variance relative to the model without covariates*.

Table 7 shows that for the vast majority of studies and contexts, a significant main effect of both familiarity and involvement was found. Specifically, a significant main effect of both involvement and frequency was found for 13 out 15 target usage situations across all product categories. By contrast, all interaction terms were not significant (*p* > 0.05) except for a single instance—Context 1 of Study 7a—where a significant 2-way interaction between involvement and appropriateness was found. A tendency toward a significant interaction between frequency and appropriateness was found in Context 3 of Study 8a (*p* = 0.062).

**Figure 5:**
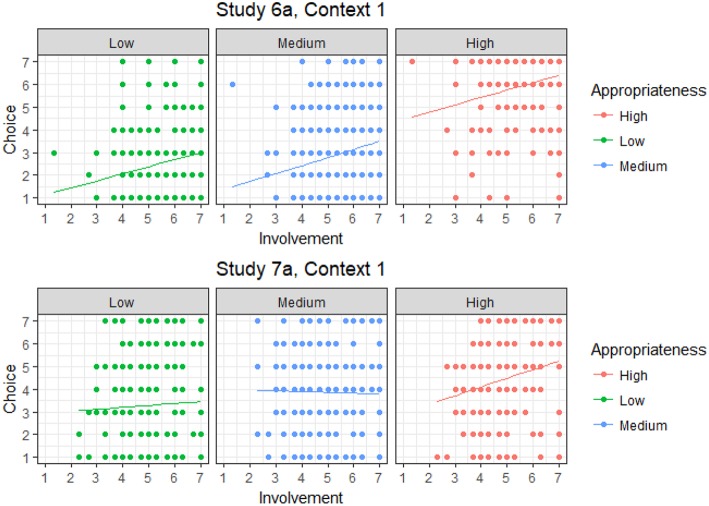
ANCOVA plots showing the effect of product involvement on choice for different situational appropriateness level. The top plot shows an instance of a main effect (Study 6a, Context 1), which was observed in the vast majority of case. The bottom plot shows the only instance of significant interaction (Study 7a, Context 1).

Since interactions were overwhelmingly non-significant, the results presented in Table 7 clearly indicate that product involvement and familiarity do not moderate the effect of appropriateness on choice. The consistency with which main effects were found instead suggest that these traits exert an independent effect on choice. The directions of these effects were in line with the nature of these two constructs, as product involvement and familiarity were consistently associated with higher choice likelihood. By way of example, Figure 5 shows two ANCOVA plots. The upper plot shows the results from Context 1 of Study 6a, which shows a baseline effect for each level of appropriateness, consistent with the main effect reported in Table 7, but the slopes of the regression lines for involvement are essentially identical across the three appropriateness levels. The bottom plot reports the only instance of a significant interaction effect (Context 1 of Study 7a), where the effect of involvement on choice was dependent on appropriateness level. However, as already mentioned, this was the only instance in which a significant interaction was observed, whereas the upper plot in Figure 5 was observed for the vast majority of contexts and product categories. Therefore, our hypothesis that considering consumer product involvement and consumption frequency would moderate the effect of appropriateness on choice (*H*_3_) was not supported by the data.

An important insight from Table 7 pertains to the amount of variance in choice likelihood accounted for by the two covariates. This was, in general, very low, as neither product involvement nor consumption frequency accounted for significantly more variance than just appropriateness level by itself. This can be readily ascertained by looking at the last column in Table 7, where marginal gains (Δ*R*^2^) obtained by including each covariate in the ANCOVA models were in the 0−10% range for product involvement, and in the 0−4% range for consumption frequency (*Mean* = 1.2). Effect sizes for product involvement ($\Delta R_{Mean}^{2}=4.3$) were always larger than for consumption frequency ($\Delta R_{Mean}^{2}=1.2$), indicating that the former had a larger influence on choice likelihood. An interesting aspect of these results was that the effects varied quite substantially across different product categories, as well as across different usage situations within the same product category. Tentatively, larger effect sizes were obtained in “niche” usage situations, such as eating chocolate *When walking/hiking* (Context 2 of Study 9a, Δ*R*^2^ = 7%) or kiwifruit *As a digestive aid* (Context 2 of Study 10a, Δ*R*^2^ = 10%), than in more generic ones, such as drinking wine *For a special occasion* (Context 2 of Study 7a, Δ*R*^2^ = 0%) or beer *At a pub* (Context 1 of Study 8a, Δ*R*^2^ = 3%), which would seemingly fit well with the nature of the involvement construct: familiar usage situations would reduce the influence of involvement, whereas more specialized usage may imply a specific interest in the product which underpins involvement.

## 5. Discussion

The overall goal of this research was to investigate the relationship between situational appropriateness and product choice for food and beverages. This study contributes to the literature on food-related consumer behavior in at least four ways: (i) by focusing on how situational appropriateness influences food choice specifically (as opposed to acceptability or intake), (ii) by quantifying the strength of this relationship, (iii) by considering the role of individual moderators, and (iv) by providing method–independent results based on a very large and diverse sample of consumers, products, and usage contexts.

In line with our expectations, evidence across all 15 studies indicated that perceived appropriateness—i.e., the degree to which consumers associate a product with an anticipated usage situation—predicts consumers' food and beverage choices (*H*_1_). This is in line with the compatibility principle according to which usage context can acts as frame of reference to increase the salience of product characteristics that fulfill the goals associated with it (Tversky et al., 1988; Lai, 1991; Ratneshwar and Shocker, 1991; Tversky and Simonson, 1993). Importantly, this finding was very robust across different product categories, consumer samples, and usage situations. The external validity of the results is further ensured by the fact that replicating five of the studies by using a different experimental protocols (rating vs. discrete choice) resulted in nearly identical results.

In light of the coenotropic character of the situational appropriateness concept (that is, the fact they are culturally determined and acquired through one's experience) we expected both product– and individual related factors to influence the degree to which situational appropriateness would be predictive of choice. Accordingly, effect sizes were found to vary substantially both between and (to a lesser degree) within studies. With respect to moderators pertaining to variation in product alternatives explicitly considered by consumers, we considered a local adaptation of the theory of situational strength (Mischel, 1977; Chang and Tseng, 2015), and expected that large product differences in terms of perceived appropriateness would make situational appropriateness more salient to consumers, whereas smaller differences between products would result in higher variation due to personal preferences exerting a higher influence (*H*_2_). Consistent with this theoretical premise, effect sizes pertaining to the degree to which appropriateness predicted choice were found to be linearly related to the range in appropriateness spanned in the product set under study. Some degree of caution should be exerted in interpreting these results as, unlike the other two hypotheses, our approach pertaining *H*_2_ was exploratory. Since the results are technically correlational, one possibility is that appropriateness range (for which Cochran's Q is a proxy) overlapped with inter-product differences in other dimensions. Accordingly, Figures 3, 4 show that both *Q* and *R*^2^ are associated to a progressive increase of heterogeneity between products, as larger values were obtained for studies comparing different product categories compared to variants within the same product category. This was not always the case, however: for example, some of the studies where appropriateness accounted for a very high percentage of the variance in choice (Studies 9a and 9b) featured images of a chocolate bars from a single brand where the only variation was associated to extrinsic product elements (product name and packaging in this case).

This research further considered whether individual consumer traits could moderate these effects and help explain some of the variance in consumer choice left unaccounted by situational appropriateness. In particular, we considered the role of product familiarity and involvement which, on the basis of extant research (Lai, 1991; Quester and Smart, 1996, 1998; Dodd et al., 2005), were expected to dilute the importance of situational appropriateness in informing choice behavior, in favor of personal preferences and attitudes (*H*_3_). For the most part the results did not conform with expectations in this case and, unlike earlier reports (Lai, 1991), the effect of appropriateness on choice did not co–vary with either familiarity and involvement, though both traits independently exerted a positive effect on choice. This begs the question of whether there are other individual consumer traits that may possibly moderate the effect of appropriateness on choice. Given the nature of appropriateness, it is possible that personal traits that more closely relate to individuals' tendency to follow cultural norms on what is appropriate to eat in a specific situation, such as tendency to conform (Goldsmith et al., 2005), may be more relevant than product–related traits. Support for this idea was obtained in a related investigation (Jaeger et al., 2019) in which we explored inter–individual variation in product evaluations of situational appropriateness for consumption in main daily meals across a large consumer sample. The results showed that consumers could reliably be differentiated in two segments: a situationally “conforming” segment that strictly follows to common norms about what is appropriate to eat and drink at different mealtimes, and an “adaptive” segment that considers a wide range of products as appropriate for the same situations—with the former scoring higher in general tendency to conform. It would therefore seem plausible that consumer's stable propensity to relate to cultural norms would affect the degree to which appropriateness is predictive of choice.

### 5.1. Managerial Implications

In the food and beverage industry, hedonic responses have traditionally been the primary product performance indicator in CLTs. With increasing recognition that individual preferences are an insufficient basis to predict product performance in the marketplace, efforts to measure consumer responses in context are poised to increase significantly in the near future (Jaeger and Porcherot, 2017).

In the context of applied product development, the research presented in this paper consistently showed that appropriateness is predictive of consumers' food choices, indicating that this aspect should be considered as an important product performance criterion for CLTs. Evidently, a product deemed appropriate for a given situation (e.g., cereals for breakfast) may still be rejected if it is not liked, so clearly these two measures—preferences and appropriateness—should be considered in conjunction to understand and predict food choices. Though we need additional knowledge to formulate best practices on how to optimally combine these measures (for instance, a better understanding of the relationships between liking and situational appropriateness as well as the precise range of product variation which consumers can differentiate on the basis of appropriateness), this research clearly indicates that product understanding cannot be complete without appropriateness for use evaluations, as also emphasized by Jaeger et al. (2017); the IBU approach adopted in this study provides an easy and flexible way to gather information on situational fit under CLT conditions. Our results are also in agreement with previous research demonstrating the meaningfulness of situational appropriateness for differentiating stimuli within the same product category (Raats and Shepherd, 1991; Lähteenmäki and Tuorila, 1997; Giacalone et al., 2015), and thus its suitability as product performance indicator in commercial food product development, where different formulations are typically tested to identify the most promising one(s) to reach go/no go decisions on whether to continue development or move on to launch.

Besides product development, our results may also be relevant in the context of retailing. Usage situations determine the intensity and nature of the competition facing specific food and beverage products (Ratneshwar and Shocker, 1991), and can be used by retailers to inform shelf space management. For example, situational appropriateness data may be used to arrange products around intended situational benefits (e.g., a reduced alcohol wine in the health food section rather than in the wine rack)^3^, thus increasing the likelihood that they will capture consumer's attention. Additionally, this could be used to enhance the situational appropriateness for novel foods and beverages, for which consumers often find challenging to envisage appropriate usage situations (Giacalone and Jaeger, 2016).

### 5.2. Limitations and Future Research

This research found that consumers are more likely to choose situally appropriate product when making choices with a specific usage situation in mind. Though the results presented span across several product categories, they are limited to one particular methodological framework for contextual elicitation—the IBU approach (Schutz, 1994; Cardello and Schutz, 1996). A limitation of this approach is its nomothethic character, that is, one set of usage contexts is evaluated by all consumers, with the implicit assumption that all usage contexts are relevant to all consumers, which may not be the case. Additionally, the level of immersiveness of the imagined contexts may not be very high (Giacalone, 2019), although we tried to mitigate this problem by having consumers describe the situation in writing prior to any choice task. It would be interesting, therefore, to continue this line of work with ideographic methods where consumers are free to develop their own set of usage situations, such as the Repertory Grid (Fransella et al., 2004).

An additional limitation concern the type of stimuli used in this research. All studies used either product names or product images. Although these were considered a valid alternative to actual products, and have been employed effectively in previous research on the same topic (Cardello and Schutz, 1996; Creusen and Schoormans, 2005; Jaeger et al., 2005; Sester et al., 2013; Giacalone et al., 2015), an interesting question for future research is whether these results extend to consumers' evaluation of food and beverage that strongly depend on sensory modalities, such as smell and taste, which would typically be the case in CLT evaluations of food and beverages. On a related note, future studies should evaluate the importance of perceived appropriateness relative to other relevant characteristics of food and beverages, such as liking, sensory characteristics, packaging design, nutritional information, and price points. Studies adopting systematic variation in a conjoint analytical framework, where usage contexts can be specified as design factors of interest—as demonstrated e.g., in Jaeger et al. (2001) and Jaeger and Rose (2008)—would be especially beneficial.

Lastly, with respect to individual consumer traits, additional research is required to better understand the antecedents of perceived situational appropriateness and how these underpin the effect of appropriateness on choice. Future research on consumer segmentation based on appropriateness data should seek to elucidate possible relationships between relevant demographic, psychographic or behavioral variables. It is also worth noting that in this research we have considered the notion of “culture” as unambiguously shaping consumers' relationship to food within the same nation/ethnicity. In reality, however, it is likely that specific subgroups within the same culture differ systematically in the foods and beverages in what they regard as situationally appropriate. To the extent that this can be operationalized (e.g., in terms of attitudes—such as vegetarianism, or other psychographics—such as being a foodie), it should also constitute a meaningful avenue for understanding inter-individual variation in this domain.

## 6. Conclusion

This research has examined the role of perceived product appropriateness on consumers' choices of food and beverages. In a series of 15 studies, perceived situational appropriateness was found to predict consumer choice, a finding that was consistently replicated across multiple product categories experimental conditions. This research thus indicates that when consumers make choices with a specific usage context in mind, products that are salient on that particular dimension, that is, are perceived as more situationally appropriate, will be more likely to be chosen.

Effect sizes were found to vary substantially, and accordingly this paper has considered potential product– and individual–related moderators. Though all target products could be significantly differentiated from one another, effect sizes were found to increase linearly with the range of appropriateness in the product set under consideration. In light with the theory of situational strength, this finding suggests that when product differences are small, consumers may tend to choose according to their own existing preferences and personality, whereas when differences are large cultural norms regarding appropriateness become more salient and co-inform choice behavior. The study also considered the potential moderating role of consumers' familiarity and involvement with a specific food and beverage category. Although both variables were found to significantly affect (increase) choice likelihood, the effect of appropriateness on choice did not co–vary with either of them.

Future research is advised to address questions left unanswered in this work, such as the relative importance of situational appropriateness in relation other common product performance indicators (e.g., liking), whether results can be extended to other types of stimuli and sensory modalities (e.g., tasting), and whether other individual traits (e.g., tendency to conform) that moderate the effect of appropriateness on choice may be identified.

## Data Availability

The datasets generated for this study are available on request to the corresponding author.

## Ethics Statement

The research was covered by a general approval from the Human Ethics Committee at the New Zealand Institute for Plant & Food Research (PFR). All participants gave informed written consent and were compensated in cash for their participation.

## Author Contributions

DG and SJ were equally involved in the conception of the work, research design, data analysis and interpretation, drafting the manuscript, and the final approval of the version to be published.

### Conflict of Interest Statement

SJ is employed by the New Zealand Institute for Plant and Food Research Ltd. The remaining author declares that the research was conducted in the absence of any commercial or financial relationships that could be construed as a potential conflict of interest.

## Footnotes

^1^For example, a value of +1 could be obtained if a respondent selected a product as MOST likely to choose two times, and selected the same product once as LEAST likely to choose. A value of zero could mean that a product was chosen as LEAST equally often as it was chosen as MOST, or alternatively that it was never selected as either MOST or LEAST.

^2^The table only reports results for Studies 6a–10a because data on familiarity and involvement were not collected in the remaining studies.

^3^This fits naturally with the concept of appropriateness that implies that product categories are defined in terms of possible usages (e.g., “as a snack,” “for breakfast”) rather than nominally (e.g., “chocolate,” “fruit”).
