# Supplementary material for: Perceived Situational Appropriateness as a Predictor of Consumers' Food and Beverage Choices
Source: Front Psychol. 2019 Jul 31;10:1743. doi: 10.3389/fpsyg.2019.01743 (PMC6685410; doi:10.3389/fpsyg.2019.01743)

**SUPPLEMENTARY MATERIAL**

To accompany paper titled: “Perceived situational appropriateness as a predictor of consumers' food and beverage choices” by D. Giacalone and S. R. Jaeger, to be published in *Frontiers in Psychology*.

**Part 1.** Mean choice likelihood ratings (7-pt scale) by level of product appropriateness for Study 1 (Breakfast items). This plot is also reported in the main document (Fig. 1).

**Part 2.** Mean choice likelihood ratings (7-pt scale) by level of product appropriateness for Study 2 (Bakery items).

**Part 3.** Mean choice likelihood ratings (7-pt scale) by level of product appropriateness for Study 3 (Seafood).

**Part 4.** Mean choice likelihood ratings (7-pt scale) by mean product appropriateness for Study 4 (Beverages).

**Part 5.** Mean choice likelihood ratings (7-pt scale) by level of product appropriateness for Study 5 (Chocolate flavour concepts).

**Part 6.** Mean choice likelihood ratings (7-pt scale) by level of product appropriateness for Studies 6a-10a. The three plots corresponding to Study 9a are also shown in the main document (Fig. 3).

**Part 7.** Mean B—W scores by level of product appropriateness for Studies 6b-10b. The three plots corresponding to Study 9b are also shown in the main document (Fig. 3).

**Part 1.**


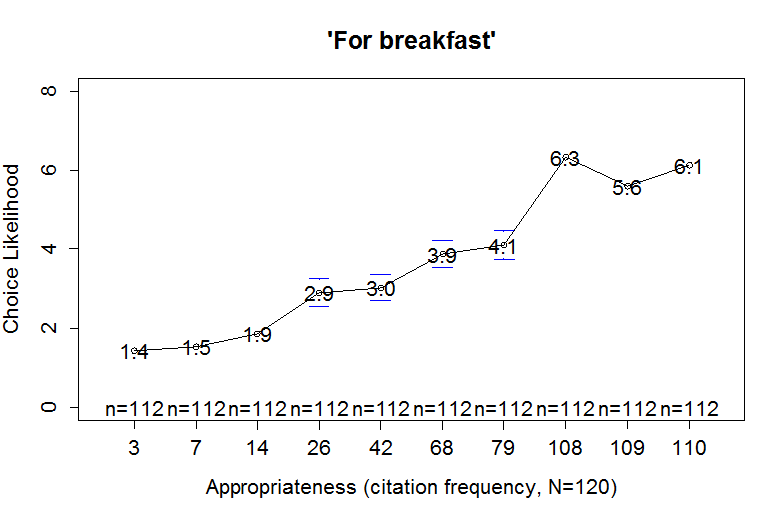


**Part 2.**


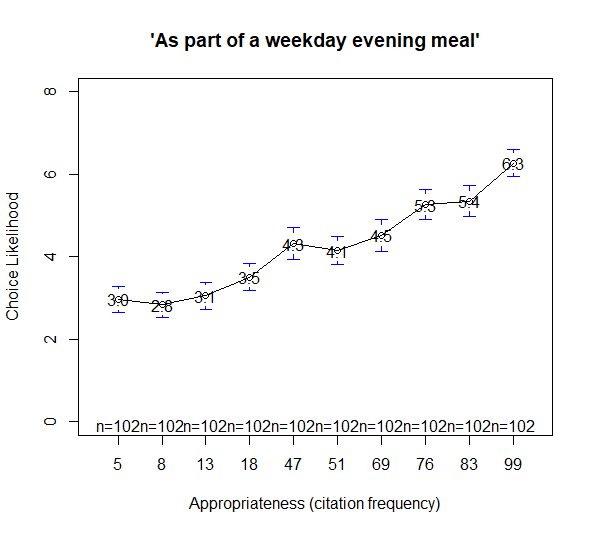


**Part 3.**


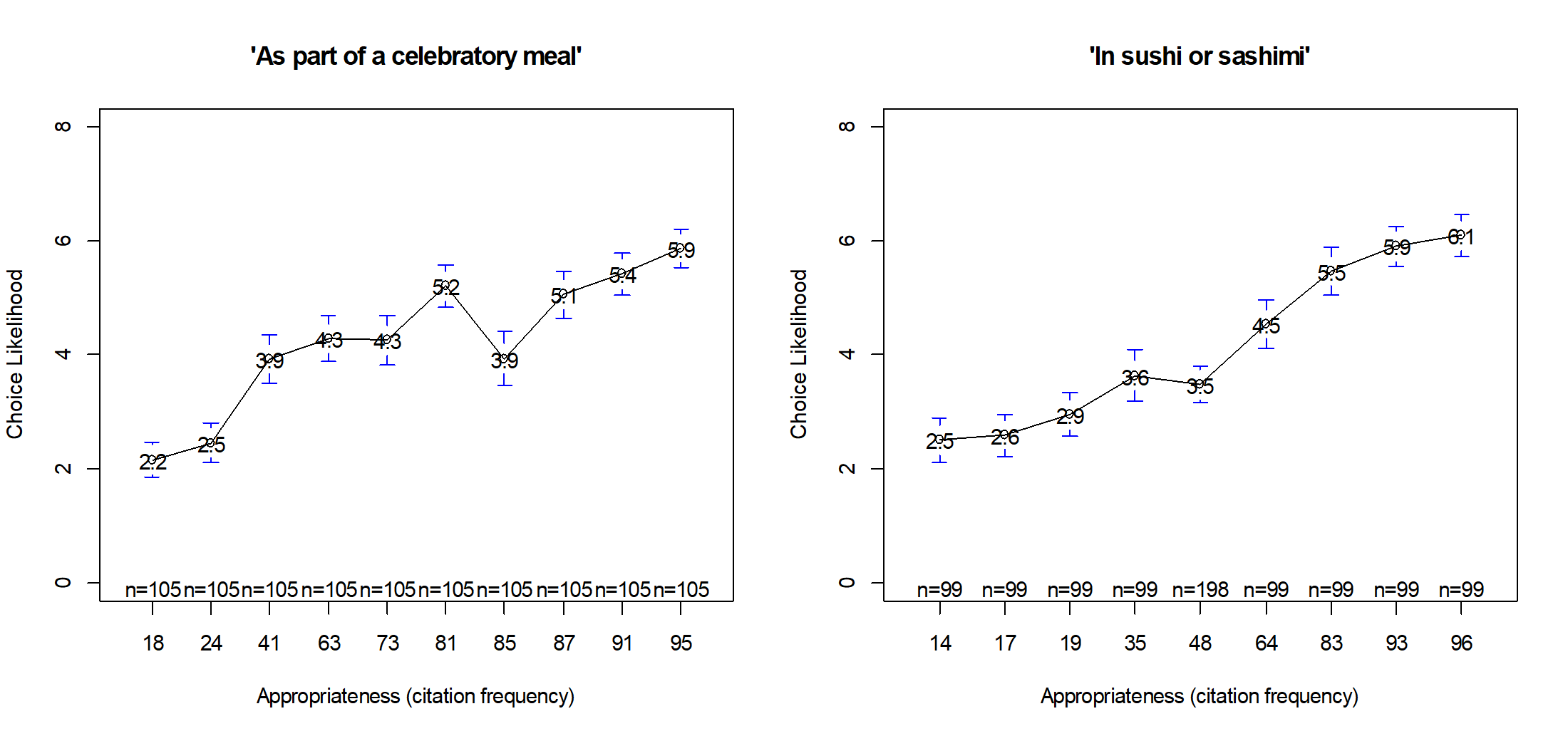


**Part 4.**


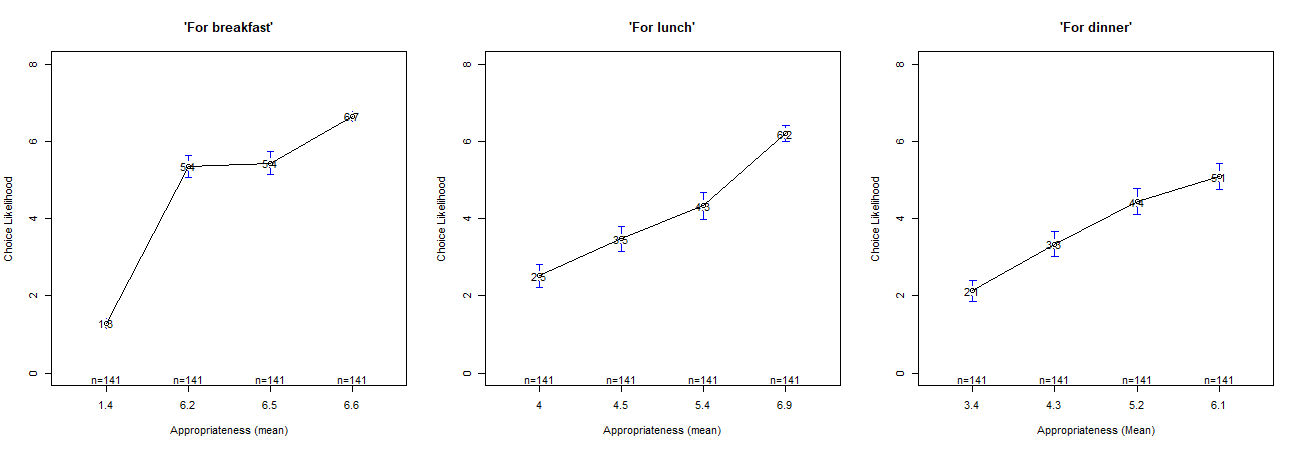


**Part 5.**


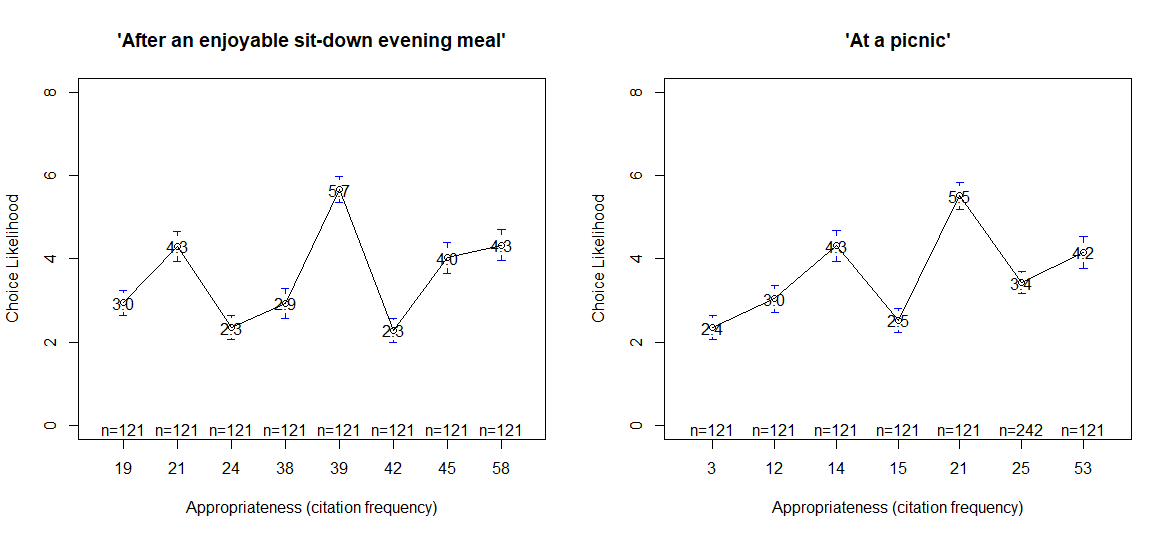


**Part 6.**


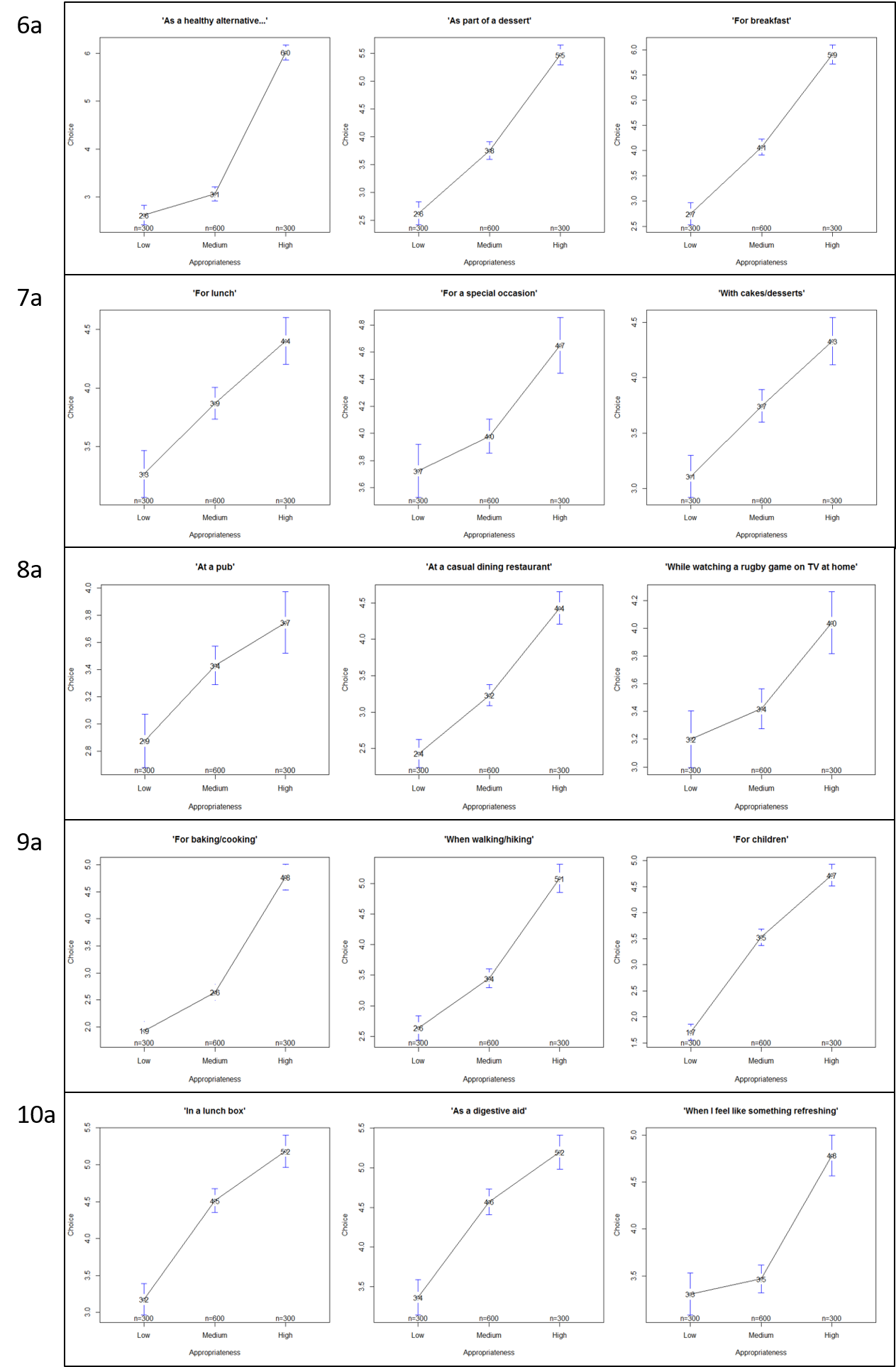


**Part 7.**


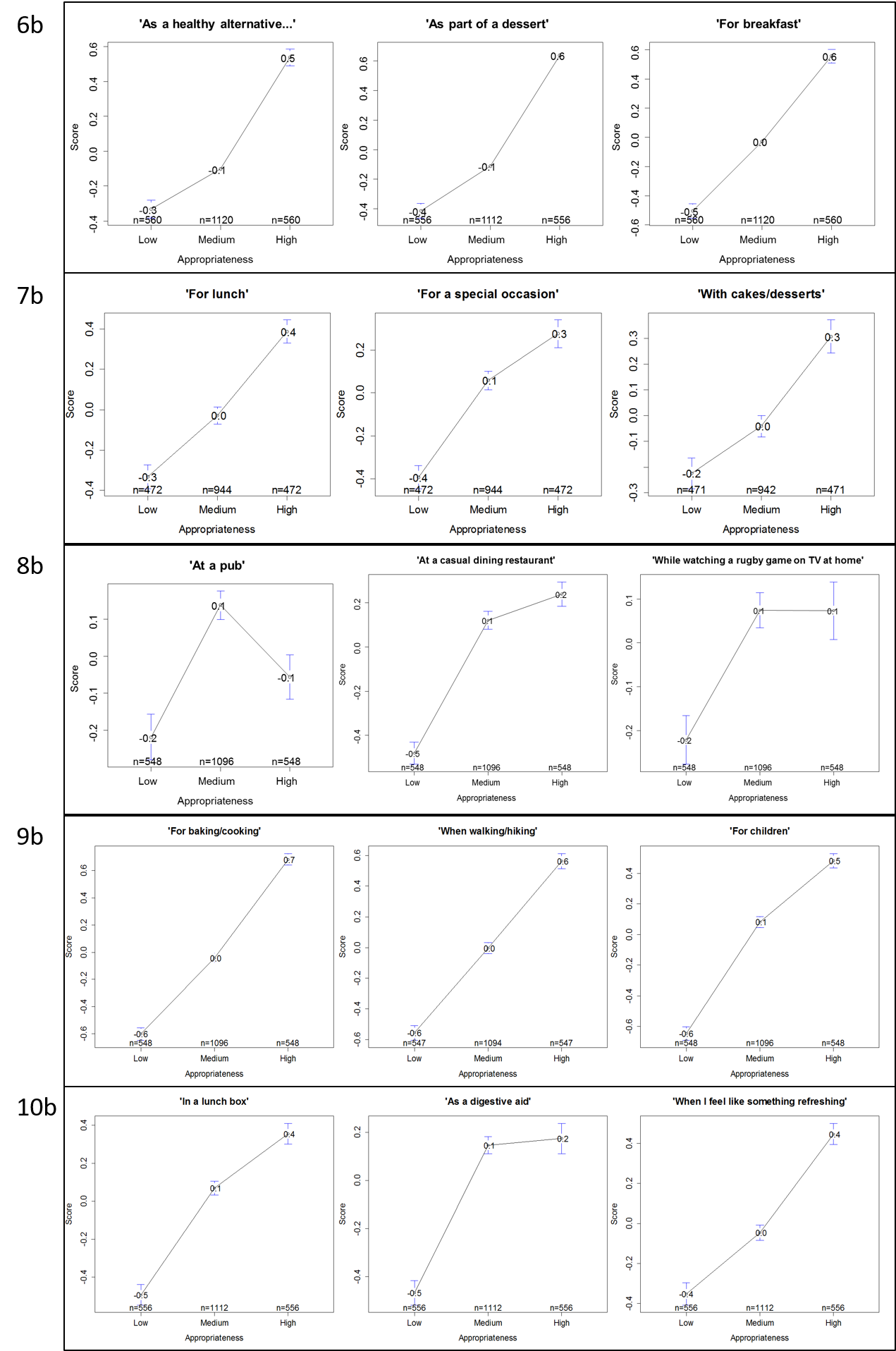

Supplement: Supplementary file 1 [file Table_1.DOCX]
